# Crowdsourcing to explore views about asthma severity among asthma experts

**DOI:** 10.1186/2045-7022-3-S1-O7

**Published:** 2013-05-03

**Authors:** Jan Lötvall, Nikos Papadopoulos, Adnan Custovic

**Affiliations:** 1 Krefting Research Centre, Sweden; 2 University of Athens, Allergy Dept, 2nd Pediatric Clinic, Greece; 3 University of Manchester, University Hospital of South Manchester NHS Foundation, UK

## 

Defining severe asthma and degrees of severity of asthma has been complicated, with classifications changing over time and also in different publications. The aim of the current investigation was therefore to start explore views about asthma severity among a wide group of asthma experts. We defined an asthma expert as an individual that since January 2005 has been an author for correspondence on a scientific publication on asthma (search term hit) in any of the top seven respiratory or allergy journals (impact factor). A brief questionnaire was distributed to 4791 e-mail addresses manually curated from the journals. Nine days after the first e-mail was circulated, and four days after a reminder e-mail, 725 individuals had responded. Of the respondents, 84.5% stated that they are either “very knowledgeable” or “knowledgeable” in asthma, and 14% state that they understand some aspects of asthma. A large majority (80.8%) of respondents suggested that reaching out to a large number of experts to crowdsource on topics related to asthma is “reasonable”, whereas 8.9% did not think so. Respondents were also asked which crucial factors contribute to asthma severity, and the responses were: 1) exacerbation frequency (84%), 2) exacerbation severity (78.4%, 3) need of extensive medication (72.2%), 4) low lung function (57.7%), 5) night-time awakenings (50.1%), 6) degree of drop in lung function when attacks occur (44.5%), 7) number of asthma symptoms (40.3%), 8) presence of any comorbidity (39.6%), 9) degree of bronchial hyperresponsiveness (39.2%), 10) lack of control according to brief questionnaire (36.8%), 11) degree of variability of lung function (36%). A small majority (57%) voted that the definition of “severe asthma” should be given to those that are uncontrolled with current conventional therapy, but an important minority (38.3%) disagreed with this definition. Outreach to a wide group of experts in the field of asthma resulted in a significant response rate, where a vast majority acknowledge themselves as experts in the field, and also thought that this approach is reasonable. Crowdsourcing may be efficient to develop wide consensus on different aspects of asthma, including a definition of severe asthma. However, additional crowdsourcing surveys, involving comments from current respondents, will be needed. This work was financed by EAACI.

